# Systematic Mendelian Randomization Exploring Druggable Genes for Hemorrhagic Strokes

**DOI:** 10.1007/s12035-024-04336-9

**Published:** 2024-07-09

**Authors:** Lun-Zhe Yang, Yong Yang, Chuan Hong, Qi-Zhe Wu, Xiong-Jie Shi, Yi-Lin Liu, Guang-Zhong Chen

**Affiliations:** 1 Department of Neurosurgery, Guangdong Provincial People’s Hospital (Guangdong Academy of Medical Sciences), Southern Medical University, Guangzhou, China; 2 Department of Neurosurgery, Guangdong Cardiovascular Institute, Guangdong Provincial People’s Hospital, Guangdong Academy of Medical Sciences, Guangzhou, China

**Keywords:** Druggable genes, Hemorrhagic strokes, Mendelian randomization, Summary-data-based Mendelian randomization

## Abstract

Patients with hemorrhagic stroke have high rates of morbidity and mortality, and drugs for prevention are very limited. Mendelian randomization (MR) analysis can increase the success rate of drug development by providing genetic evidence. Previous MR analyses only analyzed the role of individual drug target genes in hemorrhagic stroke; therefore, we used MR analysis to systematically explore the druggable genes for hemorrhagic stroke. We sequentially performed summary-data-based MR analysis and two-sample MR analysis to assess the associations of all genes within the database with intracranial aneurysm, intracerebral hemorrhage, and their subtypes. Validated genes were further analyzed by colocalization. Only genes that were positive in all three analyses and were druggable were considered desirable genes. We also explored the mediators of genes affecting hemorrhagic stroke incidence. Finally, the associations of druggable genes with other cardiovascular diseases were analyzed to assess potential side effects. We identified 56 genes that significantly affected hemorrhagic stroke incidence. Moreover, *TNFSF12*, *SLC22A4*, *SPARC*, *KL*, *RELT*, and *ADORA3* were found to be druggable. The inhibition of *TNFSF12*, *SLC22A4*, and *SPARC* can reduce the risk of intracranial aneurysm, subarachnoid hemorrhage, and intracerebral hemorrhage. Gene-induced hypertension may be a potential mechanism by which these genes cause hemorrhagic stroke. We also found that blocking these genes may cause side effects, such as ischemic stroke and its subtypes. Our study revealed that six druggable genes were associated with hemorrhagic stroke, and the inhibition of *TNFSF12*, *SLC22A4*, and *SPARC* had preventive effects against hemorrhagic strokes.

**Graphical Abstract:**

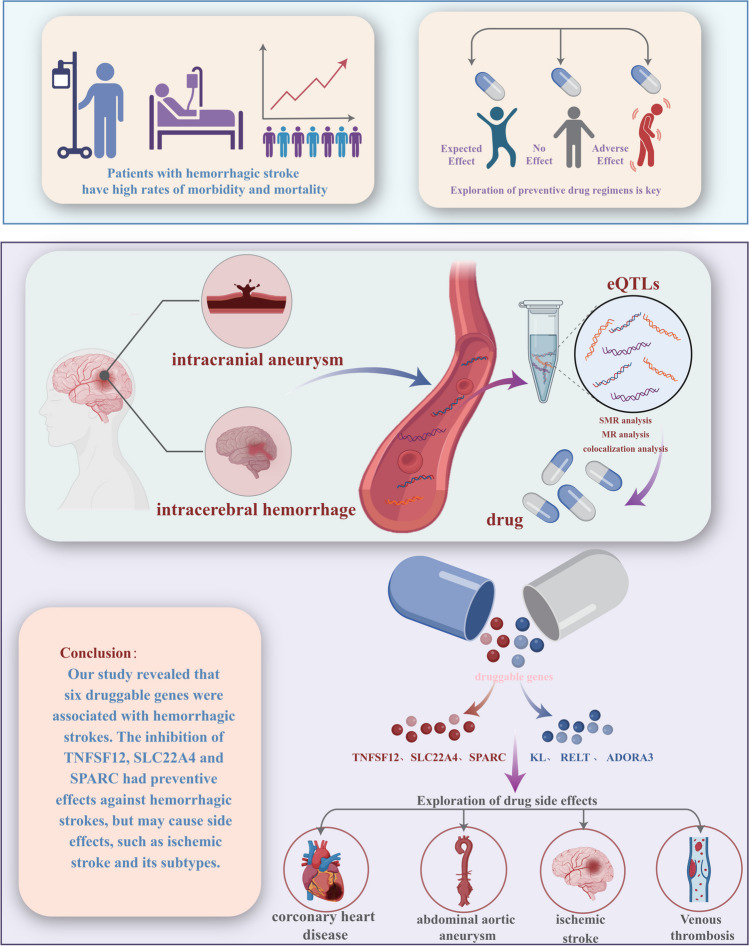

**Supplementary Information:**

The online version contains supplementary material available at 10.1007/s12035-024-04336-9.

## Introduction

Hemorrhagic strokes mainly include intracerebral hemorrhage (ICH) and subarachnoid hemorrhage (SAH), which account for approximately 13% of all strokes [1]. ICH is a tissue injury caused by the rupture of microvessels deep in brain tissue and the accumulation of blood in the brain parenchyma. Secondary brain damage from later blood clot enlargement and perihematomal cerebral edema can lead to neurological deterioration and an increased likelihood of poor functional outcomes and mortality [2]. Eighty percent of SAH cases result from the rupture of intracranial aneurysms (IAs). One-third of the patients die without admission to the hospital, accompanied by complications such as rebleeding, delayed cerebral ischemia, and hydrocephalus, which seriously affects their quality of life [3, 4]. Considering the high morbidity and mortality of hemorrhagic strokes, prevention is very important. The current treatments are mainly surgery. With the use of effective drugs for preventive therapy, the incidence of hemorrhagic stroke can be reduced while avoiding damage caused by surgery; therefore, the exploration of preventive drug regimens is key.

The majority of research on drugs for ICH prophylaxis has concentrated on lipid-lowering therapies. However, the preventive effects of these agents have been inconsistent, and multiple meta-analyses have shown no evidence of the effect of lipid-lowering drugs on ICH [5, 6]. Observational studies on SAH suggest that antiplatelet agents may confer protective effects by decreasing the incidence of IA rupture [7, 8]. Moreover, different types of antihypertensive drugs have different effects on IA, with angiotensin-converting enzyme inhibitors and beta-blockers reducing the risk of IA, while calcium channel blockers have opposite effects; however, studies have shown that antihypertensive drugs are not associated with IA [9, 10]. In observational research, these findings are always constrained by the risk of confounding bias.

The rapid development of genomics has increased the prospects for the development of novel drugs. The development of drugs with genetically supported targets has a significantly higher success rate and a much lower rate of attrition [11]. Mendelian randomization (MR) is an epidemiological approach based on genome-wide association studies (GWASs), and it has been increasingly used for the discovery and validation of drug targets [12]. Single-nucleotide polymorphisms (SNPs) within a certain range near the target, such as expression quantitative trait loci (eQTLs) and protein quantitative trait loci (pQTLs), that have a significant effect on the corresponding product are used as instrumental variables (IVs) in drug target MR analysis research and are analyzed with disease GWAS datasets to determine whether the targets have an effect on the disease at the genetic level [13]. Drug target MR analysis has been performed for individual drugs for hemorrhagic stroke treatment, but no study has comprehensively analyzed potentially druggable genes associated with hemorrhagic stroke.

Accordingly, in this study, we used the eQTLs identified in blood and performed MR analysis with GWAS datasets of SAH and ICH to systematically identify effective druggable genes.

## Methods

### Study Design

**Fig. 1:**
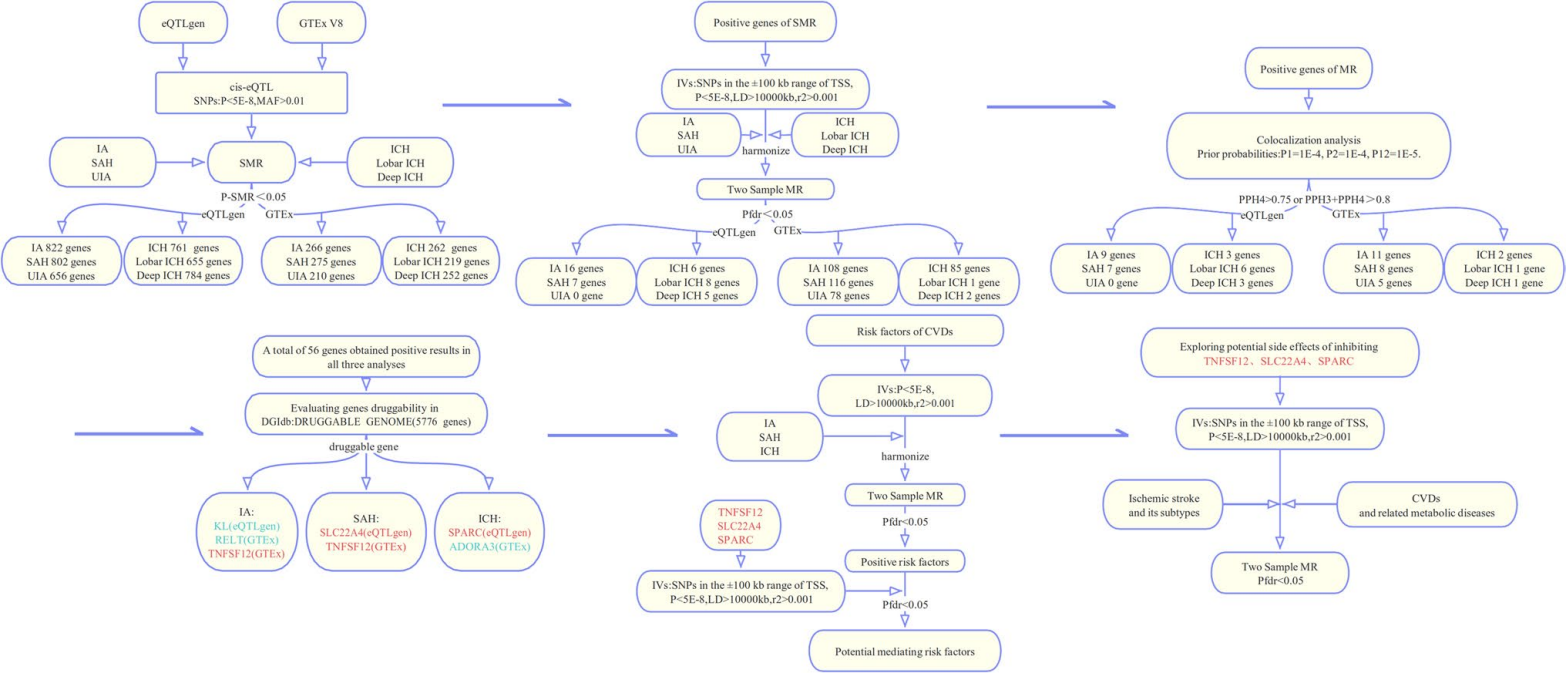
The process of our Mendelian randomization analysis. MR, Mendelian randomization; SMR, summary-data-based Mendelian randomization; eQTL, expression quantitative trait locus; IA, intracranial aneurysm; UIA, unruptured intracranial aneurysm; SAH, subarachnoid hemorrhage; ICH, intracerebral hemorrhage; CVD, cardiovascular disease; SNP, single nucleotide polymorphism; IV, instrumental variable; MAF, minor allele frequency; TSS, transcriptional start site; LD, linkage disequilibrium; *P*fdr, false discovery rate corrected *P* value; PP, posterior probability; eQTLgen, eQTLGen Consortium; GTEx, Genotype-Tissue Expression project; DGIdb, Drug-Gene Interaction Database

We performed our analysis using the following procedures: (1) preliminary screening of the targets for hemorrhagic stroke using summary-data-based Mendelian randomization (SMR), (2) further two-sample MR analysis to obtain causal associations between SMR-screened targets and hemorrhagic strokes to reduce the spectrum of positive results, (3) colocalization analysis of positive targets identified by MR analysis to verify the robustness of the results, (4) search of the Drug–Gene Interaction Database for druggable genes, (5) causal analysis of cardiovascular risk factors with hemorrhagic strokes and assessment of the effects of validated druggable targets on risk factors to explore possible mediators of targets’ effects on diseases, and (6) assessment of the effects of druggable targets on other cardiovascular diseases (CVDs) and related metabolic disorders to understand possible side effects. Our MR analysis met three major assumptions: (1) IVs were significantly linked with exposures; (2) IVs were not associated with confounders that mix exposure-outcome correlations; and (3) IVs did not act directly on outcomes but rather influenced them through exposures [14]. Further details are provided below, and the process of this study is shown in Fig. 1.

### Genetic Datasets

The eQTL datasets used in this study were obtained from the Genotype-Tissue Expression project (GTEx) version 8 and the eQTLGen Consortium. The GTEx project examined 15,201 RNA sequencing samples that were taken from 49 tissues of 838 postmortem donors (with 85% European individuals) and obtained the associated eQTL datasets [15]. The eQTLGen Consortium analyzed blood gene expression in 31,684 European individuals from 37 consortiums to identify eQTL information [16]. The cis-eQTL is located within the region of the regulated gene itself, such as the promoter and coding region of the gene, and is close to the regulated gene (within 1 Mb on either side of the coding gene). Furthermore, most drugs function through the circulation. Therefore, we used cis-eQTLs from blood samples from two consortia as our study’s IVs. For each detection, a false discovery rate of < 0.05 was considered significant [15, 16].

### IA and ICH Datasets

The SAH data were based on a GWAS meta-analysis of intracranial aneurysms from the International Stroke Genetics Consortium. Data for individuals of European ancestry were obtained from individual genetic analysis of 23 cohorts with 7495 patients and 71,934 control individuals. Patients susceptible to IA, such as Marfan syndrome, autosomal dominant polycystic kidney disease, and Ehlers‒Danlos disease, were excluded; ultimately, a total of 4,471,083 SNPs passed the quality control. When all IA patients were categorized by imaging, 69% of the patients had SAH due to ruptured IAs (5140 patients), 28% had unruptured IAs (UIAs) (2070 patients), and 3.8% had an uncertain rupture status [17]. In our study, overall IA data, UIA data, and SAH data were analyzed in a stratified manner.

The data for ICH were based on a GWAS meta-analysis of 3026 individuals (1545 patients and 1481 control individuals) of European ancestry, with the diagnostic criteria being new onset of acute neurological deficit and the presence of intracerebral parenchymal hemorrhage on imaging; in addition, patients with trauma, tumors, vascular malformations, or secondary hemorrhage were excluded [18]. According to our stratified analysis, ICHs were grouped into lobar ICHs (684 patients; lobar ICHs mostly occurring in the cerebral cortex or cortical-subcortical junction) and deep ICHs (1161 patients; deep ICHs mostly occurring in the deeper parts of the hemisphere, cerebellum, and brainstem).

### Identification of Possible Positive Targets via SMR Analysis

We first used the SMR method as a preliminary analytical tool, which integrates and analyzes GWAS data with eQTL data to identify causal associations between gene expression levels and complex traits [19]. We used SMR software, version 1.03, developed by Yang Lab of Westlake University, to analyze all eQTL data within eQTLGen and GTEx and the heterogeneity in dependent instrument (HEIDI) test to detect the presence of linkage disequilibrium (LD). The threshold for filtering SNPs was the standard of 5 × 10^−8^ and MAF > 0.01. The results were considered positive at *P* values < 0.05. Detailed descriptions of the software and its data parameters were obtained from https://yanglab.westlake.edu.cn/software/smr/#Overview.

### Further Screening Using Two-Sample MR Analysis

If there are two different causal variants with a high degree of LD, the SMR may not have the statistical power to identify pleiotropy from linkage, leaving the result unstable [19]. We further performed two-sample MR analysis on the positive results obtained from SMR analysis. The R package Two Sample MR V.0.5.7 was used to perform the analysis. First, cis-eQTLs of positive genes were extracted as IVs, with the criterion of SNPs with *P* values < 5 × 10^−8^ and within ± 100 kb from the transcriptional start site of each gene [15, 16]. The window for LD was set to 10,000 kb with *r*^2^ < 0.001 (based on the 1000 Genomes European Reference Panel) to assure independence [20]. We also removed SNPs that could not be matched or had palindromic sequences when harmonizing the gene eQTL data with the outcome GWAS data.

The Wald ratio method was selected as the primary analysis method when only a single SNP was available, and the primary analysis method was the inverse-variance weighting (IVW) method if there were multiple SNPs. The results are expressed as the disease odds ratio (OR) per 1-SD change in gene expression. MR‒Egger regression, the weighted median, and the weighted mode were used as supplementary methods for sensitivity analysis [21]. When the number of SNPs exceeded 2, the presence of horizontal pleiotropy was determined by whether the MR‒Egger intercept was close to 0 [22]. The use of Cochran’s *Q* statistic to assess the presence of heterogeneity can also help assess horizontal pleiotropy, with *P* values < 0.05 indicating the presence of heterogeneity. In addition, leave-one-out analysis was performed by removing each SNP alone to assess the robustness of the MR results. The results were corrected by multiple comparisons using false discovery rate (FDR) correction to reduce the false-positive rate, and FDR-corrected *P* values < 0.05 were considered to indicate positive results.

### Colocalization Analysis

Colocalization analysis is used to determine whether two phenotypes share the same causal variant within a region, which can provide strong evidence of overlapping genetic mechanisms between the two phenotypes [23]. There are five hypotheses: within the region analyzed via colocalization, H0: there is no SNP associated with exposure or outcome; H1/H2: there are SNPs associated with either exposure or outcome; H3: there are SNPs associated with both exposure and outcome but as two distinct SNPs; and H4: there are SNPs associated with both exposure and outcome and as the same SNP. We performed colocalization analysis between genes that were significant in both MR analyses and hemorrhagic strokes using the R package COLOC V.5.2.3 and default prior probabilities of *P*1 = 1 × 10^−4^, *P*2 = 1 × 10^−4^, and *P*12 = 1 × 10^−5^. *P*1, *P*2, and *P*12 represent the probabilities that a SNP is significantly associated with eQTLs, hemorrhagic strokes, or both, respectively. The posterior probability (PP) and likelihood of the five hypotheses were calculated using the Bayesian test, and the results were visualized using LocusCompareR [23, 24]. A range of genes ± 500 kb was taken as the region for colocalization analysis. We considered a significant colocalization analysis result of PPH4 > 0.75 or PPH3 + PPH4 > 0.8; genes strongly colocalized with hemorrhagic stroke were considered potential targets.

### Assessing the Druggability of Genes

The Drug–Gene Interaction Database (DGIdb V.4.2.0) summarizing drug–gene interactions from existing studies and several drug–gene databases provides information on 42 potential drug categories and at least 49 interaction types, with a total of 5776 druggable genes identified [25]. We searched for positive targets obtained after multiple analyses from the DGIdb. A target was considered druggable if it was classified as part of the druggable genome in the drug information provided.

### Evaluation of Potential Mediators

We further explored the potential mechanisms through which the identified drug targets influence hemorrhagic stroke incidence. Hypertension, smoking, and alcohol consumption are the leading causes of worldwide disease burden and obviously contribute to the occurrence of CVD [26]. Diabetes mellitus and improper glucose metabolism are the main causes of CVD, and CVD, which is the leading cause of death in patients with diabetes mellitus, is the primary preventive complication. Obesity serves as an important precursor to CVD complications [27]. Furthermore, lipids play an important role in CVD by affecting inflammation, leukocytes, and cardiovascular function, and lipid-lowering drugs are often used as critical therapeutic agents for a variety of diseases [28]. Therefore, we performed two-sample MR analyses of the following cardiovascular influences on patients with hemorrhagic stroke: hypertension (GWAS data published by the UK Biobank consortium, which were analyzed by the Neale laboratory among 360,000 people of white British ancestry) [29]; blood pressure (systolic blood pressure, diastolic blood pressure)[30]; smoking and alcohol consumption (drinks per week, smoking initiation, cigarettes per day, smoking cessation, age of initiation) [31]; lipid type (high-density lipoprotein, low-density lipoprotein, very low-density lipoprotein, total cholesterol, total triglycerides, apolipoprotein A1, and apolipoprotein B) [32]; obesity-related parameters (body mass index (BMI), body fat percentage, waist circumference, hip circumference, waist-to-hip ratio) [29, 33, 34]; and glucose traits (fasting glucose, fasting insulin, 2-h glucose, and glycated hemoglobin levels) [35]. The IVs were selected with the condition of *P* < 5 × 10^−8^, and SNPs with *r*^2^ > 0.001 and within a 10,000-kb window were removed. The final results after FDR correction were considered positive at *P* < 0.05. Because hypertension is a well-defined risk factor for SAH and ICH, we performed multivariable MR (MVMR) analysis of hypertension, systolic blood pressure, and diastolic blood pressure to determine the predominant risk factor [36]. The inclusion criteria for SNPs in the MVMR analysis were the same as those for univariate analysis. MR mediation analysis by the coefficient product method explored whether the other positive exposures had a direct effect on hemorrhagic strokes rather than affecting blood pressure completely. Finally, we used the risk factors as outcomes to detect the effect of the targets. If there is a significant effect of both the risk factor and risk factor on hemorrhagic stroke incidence, we can consider that these risk factors may be potential mediators.

### Exploration of Potential Side Effects of Validated Targets

Considering that the danger of ischemic strokes and their contributors have similar or opposite effects on hemorrhagic strokes, we assessed the effects of the validated targets on ischemic strokes using two-sample MR analysis. The currently largest available dataset comes from a GWAS meta-analysis of stroke and stroke subtypes by the MEGASTROKE consortium, which included 446,696 European individuals and has been used in most MR analyses studying this disease [37]. We obtained data on any history of ischemic stroke (AIS), large artery stroke (LAS), small vessel stroke (SVS), or cardioembolic stroke (CES) for analysis. Data on lacunar stroke incidence were obtained from another meta-analysis that pooled previous GWASs and genes from newly recruited patients [38]. GWAS data for transient ischemic attack were obtained from Release 9 of the FinnGen consortium [39]. In addition, to investigate other potential benefits and harms of druggable genes, we also performed MR analysis of other CVDs and metabolic-related diseases, including white matter hyperintensities [40], atrial fibrillation (AF) [41], heart failure [42], chronic kidney disease [43], type-2 diabetes [44], and thoracic aortic aneurysm [45]. Some data were obtained from the UK Biobank consortium, e.g., data on venous thromboembolism (VTE), coronary atherosclerosis, major coronary heart disease event, nonrheumatic valve diseases, pulmonary embolism, peripheral artery disease [29], while other data, e.g., data on abdominal aortic aneurysm, dissection of the aorta, dissection of cerebral arteries, nonpyogenic thrombosis of the intracranial venous system, arterial embolism, and thrombosis, were obtained from the FinnGen consortium [39]. For the results of two-sample MR analysis, we considered *P* values < 0.05 to indicate statistical significance after FDR correction, and the parameters of analysis were the same as those listed above.

## Results

### MR Analysis and Colocalization Analysis

Since most of the drugs act through blood circulation, we first performed SMR analysis using whole-blood eQTLs to conduct an initial screening of the targets, with *P*-SMR < 0.05 as the criterion for positivity. The number of positive results detected in eQTLGen were IA (822), SAH (802), UIA (656), ICH (761), lobar ICH (655), and deep ICH (784), while in GTEx, it was IA (266), SAH (275), UIA (210), ICH (262), lobar ICH (219), and deep ICH (252) (Tables S3–S14).

Subsequently, we performed two-sample MR analysis of SMR-positive targets to increase the robustness of the results, with *P*fdr < 0.05 indicating positivity, and each MR analysis method required that beta values have the same direction; otherwise, data were excluded. Finally, we identified a total of 432 positive results, including 124 IA genes (eQTLGen:16; GTEx:108), 123 SAH genes (eQTLGen:7; GTEx:116), 78 UIA genes (eQTLGen:0; GTEx:78), 91 ICH genes (eQTLGen:6; GTEx:85), 9 lobar ICH genes (eQTLGen:8; GTEx:1), and 7 deep ICH genes (eQTLGen:5; GTEx:2) (Tables S15–S20). All the results were tested by the MR‒Egger intercept test to exclude the presence of horizontal pleiotropy.

**Fig. 2:**
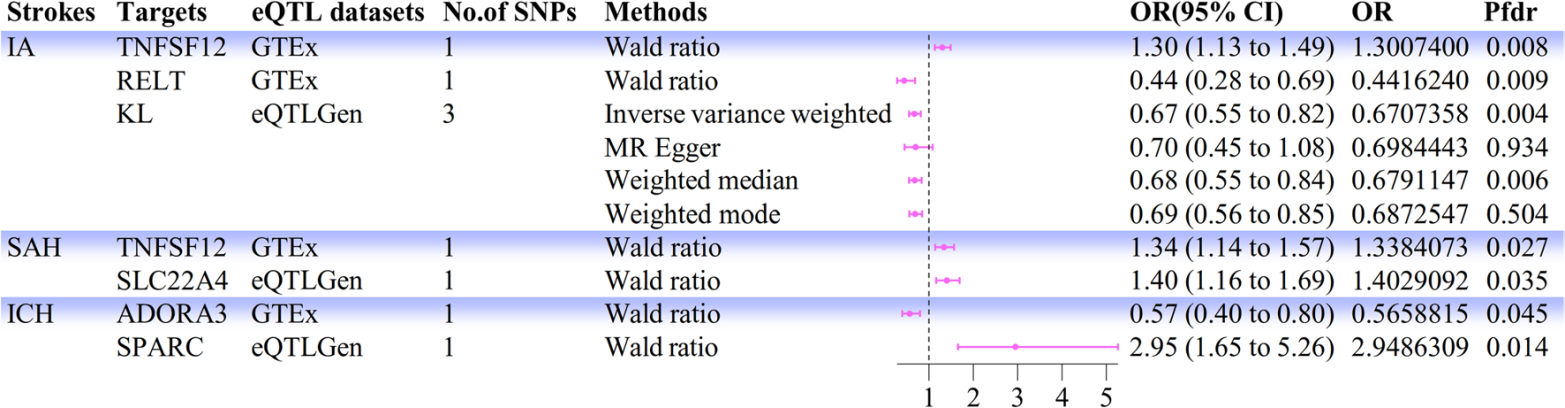
Forest plot of MR results between druggable genes and IA, SAH, and ICH. MR, Mendelian randomization; eQTL, expression quantitative trait locus; IA, intracranial aneurysm; SAH, subarachnoid hemorrhage; ICH, intracerebral hemorrhage; No. of SNPs, number of SNPs; OR, odds ratio; CI, confidence interval; *P*fdr, false discovery rate corrected *P* value

**Fig. 3:**
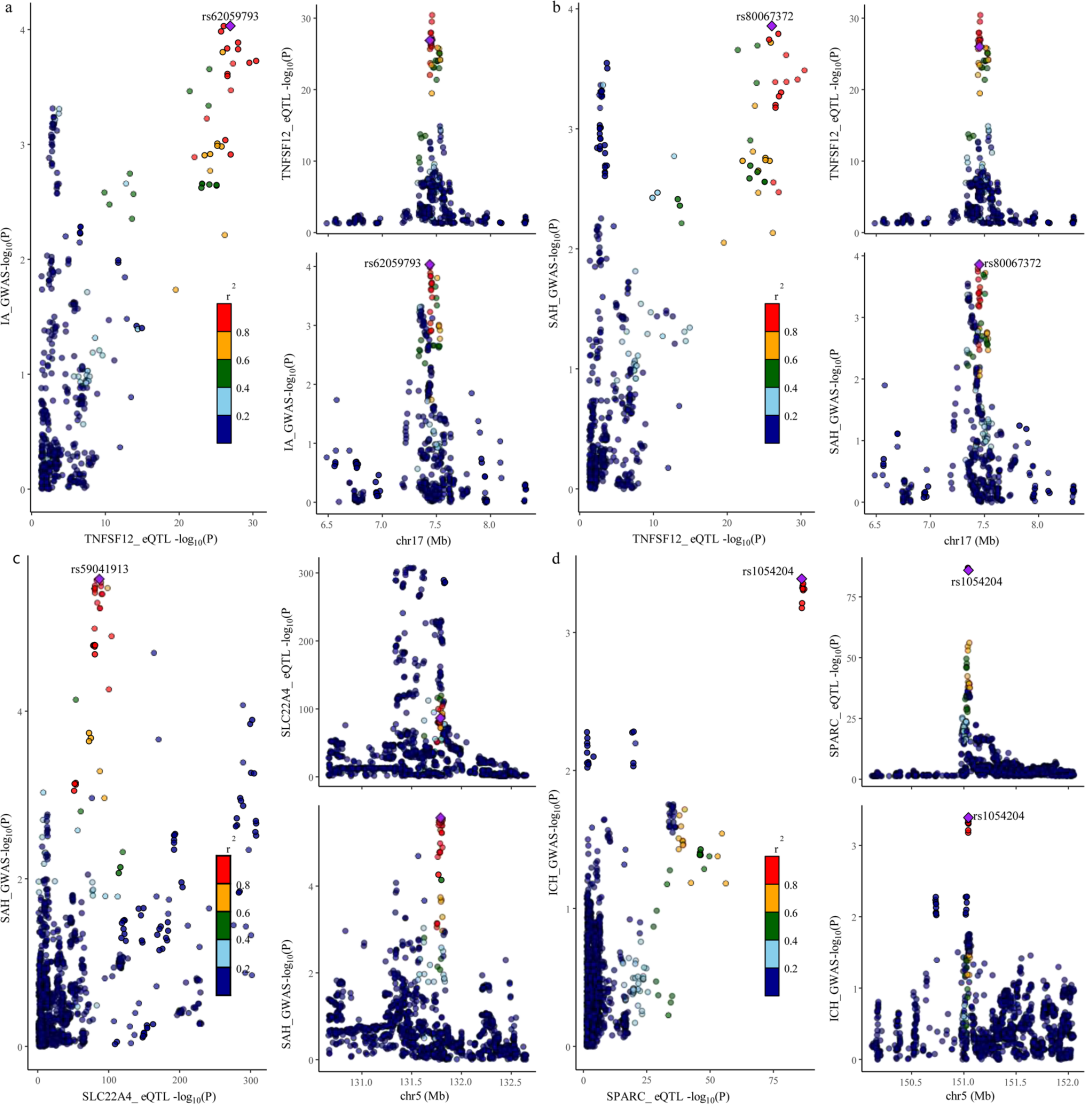
Visualizations of colocalization analysis. Colocalization analysis of **a** IA and TNFSF12; **b** SAH and TNFSF12; **c** SAH and SLC22A4; **d** ICH and SPARC. The plot on the left represents the distribution of SNPs in GWAS and eQTL at − log10(*P*); the two right plots represent the distribution of GWAS and eQTL, respectively. *r*^2^, degree of linkage between SNPs and top SNPs; GWAS, genome-wide association study; eQTL, expression quantitative trait locus; IA, intracranial aneurysm; SAH, subarachnoid hemorrhage; ICH, intracerebral hemorrhage

The 432 positive targets were subjected to colocalization analysis to clarify the likelihood of sharing causal variants. A total of 56 targets met the criteria for PPH4 > 0.75 or PPH3 + PPH4 > 0.80 (Table S21). Finally, we searched for these 56 targets in the DGIdb to obtain information on the targets, and the final results were obtained for the drug targets categorized as part of the druggable genome. After multiple screenings, we identified six druggable genes associated with hemorrhagic stroke (Fig. 2). Among those associated with IA were KL (*P*-SMR = 6.37 × 10^−4^; OR = 0.67; 95% CI, 0.55–0.82; *P*fdr = 4.12 × 10^−3^; H3 + H4 = 0.99), RELT (*P*-SMR = 1.66 × 10^−3^; OR = 0.44; 95% CI, 0.28–0.69; *P*fdr = 8.55 × 10^−3^; H4 = 0.86), and TNFSF12 (*P*-SMR = 7.87 × 10^−3^; OR = 1.30; 95% CI, 1.13–1.49; *P*fdr = 8.33 × 10^−3^; H4 = 0.88); the targets of SAH were SLC22A4 (*P*-SMR = 2.83 × 10^−3^; OR = 1.40; 95% CI, 1.16–1.69; *P*fdr = 0.035; H3 + H4 = 0.91) and TNFSF12 (*P*-SMR = 9.33 × 10^−3^; OR = 1.34; 95% CI, 1.14–1.57; *P*fdr = 0.027; H4 = 0.84); and for ICH, the targets were SPARC (*P*-SMR = 5.85 × 10^−4^; OR = 2.95; 95% CI, 1.65–5.26; *P*fdr = 0.014; H4 = 0.86) and ADORA3 (*P*-SMR = 1.61 × 10^−3^; OR = 0.57; 95% CI, 0.40–0.80; *P*fdr = 0.045; H4 = 0.77); no significant identifiable druggable gene was associated with UIA, lobar ICH, or deep ICH (Table 1). We can conclude that *TNFSF12*, *SLC22A4*, and *SPARC* may induce IA, SAH, and ICH and that blocking these targets may have a protective effect against hemorrhagic stroke (Fig. 3).

**Table 1:** Results of SMR, MR, and colocalization analysis between validated targets and hemorrhagic strokes

Strokes	Targets	eQTL datasets	SMR analysis				MR analysis (IVW/Wald ratios)					Colocalization analysis	
			Beta	se	Pval	Pval of HEIDI test	OR	95% CI	Pfdr	Q_Pval	Pleiotropy_Pval	PPH4	PPH3 + PPH4
Intracranial aneurysm	TNFSF12	GTEx	0.230	8.67E − 02	7.88E − 03	0.573	1.301	1.133 to 1.493	8.34E − 03	NA	NA	0.881	0.909
	RELT	GTEx	− 0.817	0.260	1.66E − 03	0.819	0.442	0.284 to 0.687	8.55E − 03	NA	NA	0.860	0.897
	KL	eQTL	− 0.380	0.111	6.37E − 04	3.55E − 05	0.671	0.552 to 0.815	4.13E − 03	0.704	0.874	2.65E − 05	0.999
Subarachnoid hemorrhage	TNFSF12	GTEx	0.265	0.102	9.33E − 03	0.558	1.338	1.142 to 1.569	2.73E − 02	NA	NA	0.836	0.873
	SLC224A	eQTL	0.199	6.67E − 02	2.83E − 03	7.54E − 02	1.403	1.163 to 1.692	3.52E − 02	NA	NA	8.92E − 02	0.910
Intracerebral hemorrhage	ADORA3	GTEx	− 0.569	0.181	1.61E − 03	0.431	0.566	0.401 to 0.799	4.51E − 02	NA	NA	0.767	0.776
	SPARC	eQTL	1.069	0.311	5.85E − 04	0.713	2.949	1.653 to 5.26	1.44E − 02	NA	NA	0.864	0.885

*MR* Mendelian randomization, *SMR* summary-data-based Mendelian randomization, *eQTL* expression quantitative trait locus, *HEIDI* heterogeneity in dependent instrument, *IVW* inverse-variance weighted, *OR* odds ratio, *CI* confidence interval, *Pfdr* false discovery rate corrected *P* value, *PP* posterior probability

### Mediators of Validated Druggable Genes Affecting Hemorrhagic Strokes

In analyses exploring possible mechanisms of druggable gene-induced hemorrhagic strokes, MR analysis revealed that FDR-corrected hypertension, systolic blood pressure, diastolic blood pressure, BMI, body fat percentage, and smoking initiation were associated with IA and SAH, whereas none of the risk factors were significantly associated with ICH (Table 2). Then, we performed MVMR analysis of hypertension, systolic blood pressure, and diastolic blood pressure in patients with IA and SAH to clarify which factor was predominant. The multivariate IVW results showed that hypertension (OR = 27.842; 95% CI, 1.042–743.623; *P* = 4.72 × 10^−2^) and systolic blood pressure (OR = 2.423; 95% CI, 1.361–4.312; *P* = 2.63 × 10^−3^) still had significant effects on IA, and SAH was significantly correlated with only systolic blood pressure (OR = 2.933; 95% CI, 1.519–5.664; *P* = 1.35 × 10^−3^). Although diastolic blood pressure no longer had harmful effects after adjustment (IA: OR = 0.938; *P* = 0.795; SAH: OR = 0.832; *P* = 0.515), it was excluded.

**Table 2:** Positive results of MR analysis between hemorrhagic strokes and cardiovascular risk factors

Outcomes	Exposures	Methods	No. of SNPs	OR	95% CI	Pfdr	Q_Pval	Pleiotropy_Pval
Intracranial aneurysm	Body fat percentage	IVW	196	1.799	1.365 to 2.371	1.71E − 04	1.05E − 05	0.708
	Body mass index	IVW	244	1.281	1.071 to 1.531	2.43E − 02	0.164	0.848
	Diastolic blood pressure	IVW	152	2.635	2.015 to 3.446	1.61E − 11	8.17E − 04	0.829
	Hypertension	IVW	34	370.060	46.144 to 2967.758	1.90E − 07	4.74E − 03	0.871
	Smoking initiation	IVW	33	3.035	1.717 to 5.365	5.85E − 04	0.147	0.152
	Systolic blood pressure	IVW	153	3.313	2.522 to 4.352	1.63E − 16	3.71E − 03	0.488
Subarachnoid hemorrhage	Body fat percentage	IVW	196	1.866	1.373 to 2.536	2.97E − 04	1.20E − 03	0.986
	Body mass index	IVW	245	1.314	1.063 to 1.624	4.26E − 02	9.89E − 02	0.931
	Diastolic blood pressure	IVW	152	2.814	2.078 to 3.812	2.58E − 10	5.85E − 03	0.994
	Hypertension	IVW	35	744.444	71.58 to 7742.364	2.29E − 07	1.08E − 02	0.648
	Smoking initiation	IVW	33	3.884	2.064 to 7.308	1.42E − 04	0.284	0.402
	Systolic blood pressure	IVW	153	3.779	2.767 to 5.16	1.34E − 15	1.35E − 02	0.500

*IVW* inverse-variance weighted; *No. of SNPs* number of SNPs, *OR* odds ratio, *CI* confidence interval, *Pfdr* false discovery rate corrected *P* value

For IA, when hypertension was used as a mediator, the effects of BMI (133.5%), body fat percentage (67.4%), and smoking initiation (10.6%) were mediated in part through hypertension; however, when systolic blood pressure was used as a mediator, there was no evidence that the effects of BMI, body fat percentage, or smoking initiation were mediated through systolic blood pressure. We can assume that systolic blood pressure, hypertension, body fat percentage, and smoking initiation have direct effects on IA. Moreover, for SAH patients, there was no evidence that hypertension, BMI, body fat percentage, or smoking initiation had an effect on systolic blood pressure; therefore, we believe that systolic blood pressure, hypertension, BMI, body fat percentage, and smoking initiation can directly influence SAH incidence.

Our two-sample MR analysis of druggable genes and exposures using the same parametric criteria showed that TNFSF12 was significantly associated with hypertension (OR = 1.006; 95% CI, 1.002–1.011; *P* = 6.90 × 10^−3^), which remained significant after FDR correction (IA: *P*fdr = 1.38 × 10^−2^; SAH: *P*fdr = 2.01 × 10^−2^). SLC22A4 was also causally associated with hypertension (OR = 1.01; 95% CI, 1.005–1.014; *P* = 3.46 × 10^−5^) and with SAH (*P*fdr = 1.38 × 10^−4^) (Table 3).

**Table 3:** Results of MR analysis of validated druggable genes with cardiovascular risk factors

Exposures	Outcomes	Methods	No. of SNPs	OR	95% CI	Pval	Q_Pval	Pleiotropy_Pval
TNFSF12	Hypertension	Wald ratio	1	1.006	1.002 to 1.011	6.90E − 03	NA	NA
	Systolic blood pressure		NA					
	Body mass index	Wald ratio	1	1.009	0.993 to 1.024	0.265	NA	NA
	Body fat percentage		NA					
	Smoking initiation	Wald ratio	1	1.007	0.990 to 1.025	0.422	NA	NA
SLC22A4	Hypertension	IVW	4	1.010	1.005 to 1.014	3.46E − 05	0.992	0.862
	Systolic blood pressure		NA					
	Body mass index	IVW	4	1.017	0.991 to 1.043	0.204	0.044	0.144
	Body fat percentage	IVW	4	1.013	1.001 to 1.026	3.95E − 02	0.608	0.501
	Smoking initiation	IVW	4	1.001	0.983 to 1.020	0.908	0.583	0.406

*IVW* inverse-variance weighted, *No. of SNPs* number of SNPs, *OR* odds ratio, *CI* confidence interval, *Pfdr* false discovery rate corrected *P* value

### Effects of Validated Druggable Genes on Other Diseases

To explore the potential side effects of these targets, we analyzed whether druggable genes influence ischemic stroke and found that TNFSF12, SLC22A4, and SPARC influence the occurrence of certain ischemic stroke subtypes. Moreover, TNFSF12 was shown to reduce the risk of CES (OR = 0.82; 95% CI, 0.73–0.94; *P*fdr = 0.012), and SLC22A4 was shown to reduce the risk of SVS (OR = 0.81; 95% CI, 0.69–0.96; *P*fdr = 0.036). Conversely, SPARC potentially increased the risk of AIS (OR = 1.14; 95% CI, 1.00–1.12; *P* = 0.042) and LAS (OR = 1.43; 95% CI, 1.06–1.91; *P* = 0.018), but these results were no longer significant after FDR correction. The various MR methods produced consistent effect estimates and no evidence of horizontal pleiotropy or heterogeneity.

Additionally, we analyzed other cardiovascular diseases (Table S24). Although none of the SNPs were included when TNFSF12 or AF was analyzed in two-sample MR, complementary analysis with SMR revealed that TNFSF12 was protective against AF (beta =  − 0.16; *P* = 9.80 × 10^−8^), and colocalization analysis also yielded a positive result (H3 + H4 = 0.99). Furthermore, TNFSF12 weakly increased the risk of VTE (OR = 1.002; 95% CI, 1.001–1.004; *P*fdr = 0.015). SLC22A4 acted as a risk factor for AF (OR = 1.08; 95% CI, 1.002–1.16; *P*fdr = 0.043) and increased the risk of dissection of the aorta (OR = 1.58; 95% CI, 1.12–2.23; *P*fdr = 0.029), but MR‒Egger (OR = 0.78) showed a different effect. Additionally, SPARC increased the risk of arterial embolism (OR = 1.64; 95% CI, 1.11–2.42; *P*fdr = 0.038). No significant effects of druggable genes on other diseases were found.

## Discussion

We performed a large-scale MR analysis between the GWAS data of hemorrhagic strokes and the target gene expression data (eQTL) to provide genetic support. Multiple screenings by combined MR analysis and colocalization analysis identified 56 genes associated with hemorrhagic stroke, six of which were druggable targets. Our study provides strong evidence that *TNFSF12*, *SLC22A4*, and *SPARC* may be potential risk factors for hemorrhagic stroke and that their induction of hypertension may be involved in potential pathogenesis. Inhibiting these targets offers potential to aid in preventing the development of hemorrhagic strokes. Furthermore, numerous potential side effects have been identified, such as blockingTNFSF12 possibly increasing the occurrence of both AF and CES while simultaneously lowering the risk of VTE; inhibiting SLC22A4 seemingly reducing the occurrence of AF but increasing the incidence of SVS; and inhibiting SPARC also having the potential to protect against arterial embolism and thrombosis, with no other unfavorable effects identified.

TNF superfamily member 12 (TNFSF12), also known as TNF-related weak inducer of apoptosis, is a member of the TNF superfamily that binds to its cognate receptor Fn14 and plays a role in regulating cell proliferation, differentiation, migration, apoptosis, inflammation, and angiogenesis [46]. Our study demonstrated that TNFSF12 can increase the risk of IA and SAH. MR analysis of drug targets for ischemic stroke revealed that TNFSF12 could promote SAH and ICH via the use of pQTLs to analyze blood proteins, and we obtained similar results using different IVs [47]. However, earlier investigations on the relationship of TNFSF12 with hemorrhagic stroke have been inadequate, with only one retrospective study suggesting that TNFSF12 increases hemorrhagic transformation in ischemic stroke patients; moreover, there are no data demonstrating the molecular mechanisms involved [48]. In an animal experiment, after treating mice with TNFSF12, it was shown that TNFSF12/Fn14 induced the upregulation of endothelin-converting enzyme-1 to produce more endothelin-1, and ultimately, the experimental group showed increased blood pressure and a dose‒response relationship [49]. Our study also proved that TNFSF12 can cause the effect of hypertension, while hypertension is a clear risk factor for IA, so we can assume that hypertension is an important mediator. TNFSF12/Fn14 act mainly through activation of the NF-κB pathway, which has been identified as a major factor regulating the induction of inflammatory genes in IA lesions. NF-κB activation recruits macrophages and produces proinflammatory cytokines and matrix metalloproteinases (MMPs) to break down the extracellular matrix, inducing apoptosis of vascular smooth muscle cells and degeneration of the internal elastic lamina, leading to loss of vascular wall integrity and aneurysm formation [50]. Thus, NF-κB may be an important pathway involved in this process. On the other hand, the MMP influences the development of IA through proteolytic disruption of the extracellular matrix of the vessel wall. Aoki et al. reported high expression of MMP-2 and MMP-9 within the wall of the IA [51], whereas Nuki et al. used gene knockout to determine that the incidence of IA decreased significantly in mice with MMP-9 knockout but not significantly in mice with MMP-2 knockout [52]. Another animal study demonstrated that TNFSF12/Fn14/NF-κB could increase MMP-9 activity but not MMP-2 activity [53]. Thus, MMP-9 could be a potential mediator. Finally, Fn14 is upregulated by a variety of cytokines in vessel wall damage [54], and TNFSF12/Fn14 induces the release of HMGB1 in macrophages via NF-κB, which can further activate NF-kB [55]. This vicious cycle may induce rupture of the IA, causing SAH. Similarly, the NF-κB signaling pathway mediates interactions between endothelial cells, platelets, and inflammatory responses, which disrupts coagulation-fibrinolysis homeostasis and induces deep vein thrombosis, and we hypothesize that NF-κB is also a mediator of TNFSFS12-induced VTE [56]. We also demonstrated that TNFSF12 reduces the risk of CES, whereas previous studies suggested that TNFSF12 can cause ischemic strokes [46]. The possible reason for these inconsistent results is the mediation of AF. Previous studies have demonstrated strong evidence that TNFSF12 is a potential drug target for AF and that genetic susceptibility of AF was an important etiological factor causing CES, and our study emphasizes the protective effect of TNFSF12 on AF [57]. Another reason could be the involvement of TNFSF12/CD163. Intracerebroventricular injection of soluble Fn14-Fc decoy receptor or anti-TNFSF12 antibody immediately after mouse cerebral infarction significantly reduced ischemic cerebral infarction volume and microglia activation and extent of apoptotic cell death in animal experiments [58], and the decoy receptor CD163 may block TNFSF12/Fn14 from acting and thus protect against CES. Currently, some drugs targeting TNFSF12 are undergoing preclinical experiments, such as ASP1531, L-534–0366, MRT-101, and TSCP-2. However, their primary investigational indications are mainly rheumatoid arthritis, gliomas, and lung cancers, and drugs with stroke as an indication still need to be further studied [25].

Solute carrier family 22 member 4 (SLC22A4), as well as organic cation transporter novel 1 (OTCN1), is an organic cation transporter protein. In a previous GWAS on IA, SLC22A4 was reported as a potential pathogenic gene at six unique loci [17]. Our MR analysis demonstrated that SLC22A4 can promote the developmental rupture of IAs, causing SAH. SLC22A4 is the protein that transports carnitine, choline, and levocarnitine and can generate trimethylamine (TMA) through the action of the gut microbiota. TMA is subsequently oxidized to trimethylamine-N-oxide (TMAO), and metabolomics studies have demonstrated that TMAO induces atherosclerosis and that elevated levels of TMAO can be used as a biomarker of high risk for CVD [59]. A meta-analysis that included 8 studies involving 11,750 individuals and 6176 patients with hypertension demonstrated that TMAO increased susceptibility to hypertension, with a 12% increased risk of hypertension in those with high TMAO concentrations [60]. Whereas hypertension, as an independent risk factor for SAH, may mediate the ability of SLC22A4 to cause SAH, a significant association between SLC22A4 and hypertension was also demonstrated in our study. Choline and its metabolites, such as betaine, can also increase the risk of developing CVD [59]. A nested case‒control study (*n* = 16,113) investigating the association between TMAO and stroke demonstrated a significant J-shaped association between TMAO and nonischemic stroke, but no significant associations were found for carnitine, choline, or betaine [61]. In an MR analysis of modifiable risk factors for IA, only positive correlations between IA and choline and betaine were demonstrated, but no association with TMAO was found [62]. We suggest that TMAO and its related metabolites are potential mediators of SLC22A4-related SAH, but further exploration is needed to determine which metabolites play a primary role in this process. While exploring the safety of the medication, we found that SLC22A4 can increase the risk of AF; the results of a study in which 2 prospective nested case‒control studies were conducted showed that higher choline and betaine levels were associated with an increased risk of AF [63], which supports the conclusion in our study and emphasizes the role that carnitine, choline, etc. play in SLC22A4 affecting the disease. Finally, inhibition of SLC22A4 may lead to SVS. SLC22A4, the main transporter protein of ergothioneine (ET), is responsible for uptake and reduces renal excretion, thus promoting the accumulation of ET in the body. SLC22A4 is a stable antioxidant and does not autocatalytically oxidize and generate oxygen radicals, thus protecting against chronic inflammation and reducing the risk of CVD [64]. Considering its antioxidative stress effect, SLC22A4 may reduce the incidence of SVS through this effect. Currently approved ustekinumab targeting TNFSF12 is mainly used in the treatment of inflammatory diseases such as psoriasis, Crohn’s disease, and ulcerative colitis. Whether its anti-inflammatory effect can be used for subarachnoid hemorrhage as an indication needs further experimentation [25]. In addition, drugs targeting OCTN have been used in cardiovascular and cerebrovascular diseases, and meldonium, as a carnitine analog, can inhibit the production of carnitine synthesis, which has achieved good results in the protection of ischemic stroke, and its effect on hemorrhagic stroke can be investigated in the future [65].

Secreted protein acidic and rich in cysteine (SPARC), which is also known as osteonectin/BM-40, is a matricellular glycoprotein that plays a key role in the regulation of cell–matrix interactions, tissue repair, and tissue calcification [66]. Our study identified SPARC as a risk factor for ICH and confirmed that SPARC has the potential to increase the risk of arterial embolism. There are more than ten drugs known to target SPARC, and the marketed drugs include tizanidine hydrochloride, baclofen, and NAB-PACLITAXEL [25]. Tizanidine hydrochloride and baclofen are mainly used in multiple sclerosis, but also for the relief of poststroke myotonia. Tizanidine hydrochloride and baclofen are mainly used in multiple sclerosis, but also for the relief of myasthenia gravis after stroke, and stroke remains an investigational indication for these drugs because inflammation caused by multiple sclerosis impairs the normal physiological functioning of vascular endothelial cells, which is strongly associated with hemorrhagic stroke [67]. PARC is closely associated with inflammatory responses and oxidative stress in tissues. The conversion of anti-inflammatory macrophages to proinflammatory macrophages can predispose patients to intravascular plaque formation and further recruit monocytes [68], and animal experiments have demonstrated that SPARC deficiency delays macrophage infiltration and proinflammatory cytokine expression [69]. SPARC also increased the apoptosis of vascular smooth muscle through the mitochondrial pathway while promoting the production of MMP, thus causing rupture of the internal elastic lamina of the vascular wall. Tan et al. also demonstrated that SPARC can upregulate the expression of NOX4 through TGF-β1, which contributes to the induction of an inflammatory phenotype transformation and apoptosis in smooth muscle cells, thereby worsening vascular wall damage [66, 70]. SPARC-mediated inflammation and vessel wall damage may be responsible for the development of ICH and arterial embolism and thrombosis. Another study collected cerebrospinal fluid specimens from 27 patients with cerebral amyloid angiopathy and reported that platelet-derived growth factor (PDGF)-BB was positively correlated with the titer of anti-Aβ antibody; moreover, PDGF-BB might be involved in smooth muscle cell repair and thus have a vasoprotective effect [71]. Raines et al. reported that SPARC could interact with PDGF-BB and inhibit its binding to the receptor to exert a vasoprotective effect and that its expression was more frequent in injured vessels than in control vessels [72], which might mediate the occurrence of ICH. Finally, SPARC is an adipocyte-secreted proinflammatory adipokine, and chronic inflammation in adipose tissue caused by its elevation is an important marker of obesity. SPARC also causes insulin resistance and is positively correlated with BMI, waist circumference, fasting blood glucose, and fasting insulin; therefore, SPARC may be a key participant in obesity and type-2 diabetes mellitus [73]. All of these factors are important in the pathogenesis of CVD. Our study concluded that these factors are not mediators of the effect of SPARC on ICH incidence.

In addition to the three druggable genes that may contribute to hemorrhagic stroke, we also identified three protective targets. Klotho (KL) is an antiaging gene expressed mainly in the kidney that stimulates the production of various antioxidant enzymes by increasing the activation of FoxO and signaling via the Nrf2 pathway and inhibits inflammatory pathways such as the TGF-β and NF-κB pathways [74], which may play a protective role against IA. A lack of KL leads to hyperphosphatemia and hypercalcemia. MR analysis also revealed a positive correlation between blood calcium levels and IA [75]. Wang et al. showed that KL gene deficiency leads to hyperphosphatemia by inducing KL in mice, after which hyperphosphate-induced or NF-κB-mediated MMP-2/9 can cause abdominal aortic aneurysm [76]. Considering that abdominal aortic aneurysm, an extracranial subtype of IA, has some similarities with the pathogenesis of IA, we hypothesized that KL deficiency may influence IA, which is consistent with our findings. The RELT TNF receptor (RELT) is a member of the TNFR superfamily that is expressed mainly in blood lymphoid tissues. This protein has proinflammatory effects and activates NF-κB, as well as apoptosis. There is a lack of correlation between RELT and cardiovascular disease, and the mechanism by which it protects against ICH is unknown. The adenosine A3 receptor (ADORA3) is an adenosine receptor that is broadly distributed in immunological and inflammatory cells and participates in the regulation of the central nervous, cardiovascular, peripheral, and immune systems as an important regulator of inflammatory responses. In addition to its anti-inflammatory effects, adenosine has been associated with vascular wall repair and protection, but it acts mainly through ADORA1/2, and it is unclear whether ADORA3 is involved. ADORA3 has been found to alleviate dysfunction caused by cerebral ischemia and brain injury; however, its effect on ICH has not been explored [77]. HU-010 targeting ADORA3 is undergoing a phase III clinical trial in the treatment of stroke as an indication [25].

Stroke is the second leading cause of death and the third leading cause of disability, and the global burden of stroke has increased dramatically, not only because of population growth and aging but also because of the dramatic increase in exposure to important risk factors, so prevention is always key [78]. Drugs that prevent hemorrhagic stroke, if available, may offer benefits for long-term protection and perioperative management of high-risk patients. Since the success rate of drugs development with gene-supported targets is twice as high as that of drugs without gene-supported targets, the primary objective of this study is to provide genetic evidence for drug development in hemorrhagic stroke in order to maximize drug efficacy, reduce drug toxicity and side effects, and reduce healthcare costs and to achieve precision medication and precision medicine. Our study provides the first comprehensive exploration of targets involved in SAH and ICH using eQTL data to provide genetic support for hemorrhagic stroke drug development and is the first to report that TNFSF12, SLC22A4, and SPARC have preventive effects on IA, SAH, and ICH. Despite the lack of an association between such targets and hemorrhagic strokes in previous studies, our study expands the horizon for future research. Most of the data used for the study came from the latest and largest GWAS. We performed two MR analyses and colocalization analyses to more clearly locate the targets, and only those targets with positive results after three screenings were included. The results were also corrected for FDR and tested for heterogeneity and horizontal pleiotropy to ensure the robustness of the results and to reduce the false-positive rate. Furthermore, the low repeatability of the eQTLGen data with the GTEx data, about 14%, and the presence of a 15% non-European population in GTEx may led to different results in different databases for these targets. We explored the two major databases to reduce the omission of valid targets while setting strict inclusion criteria [15, 16]. Meanwhile, eQTLGen has a larger sample size resulting in stronger evidence for the results relative to GTEx. We limited the targets to druggable genes, which can reduce the attrition rate associated with the development of drugs and improve the therapeutic efficacy for hemorrhagic stroke treatment. Our study also re-emphasizes the harmful role of hypertension. Considering the safety of the drug, we analyzed the targets associated with other CVD and showed that inhibiting targets that are harmful to hemorrhagic stroke patients may increase the risk of certain ischemic stroke subtypes and that attention should be given to the detection and the prevention of related side effects if these drugs are used in the clinic.

However, there are several limitations in our MR analysis. One of the key limitations is that MR analysis only provides evidence at the gene level, and the results usually indicate the effect of a relatively high drug dose on the disease over a short period of time, and therefore, the effect of the drug on the organism may be different in practical applications. At the same time, in order to ensure that the IVs were strongly correlated with exposure, we set the *P* threshold at 5 × 10^−8^ and the LD window was also very strict in order to ensure the independence of the IVs. As a result, many targets with only one SNP could only be subjected to the Wald ratio method and could not be tested for horizontal pleiotropy and heterogeneity, but the strict screening criteria resulted in high test power *F* and smaller variance *R*^2^ for the SNPs we analyzed (Table S25), indicating that our results are still robust and confident [79]. Furthermore, the data in this study were limited to European-ancestry individuals. Cross-ethnic genome-wide association studies have shown that many of the loci identified in European populations can be reproduced in non-European populations, suggesting broad cross-ethnic genetic similarities [80]. But the genetic unknowns of minority groups prevent the sharing of valid genetic information, and we cannot rashly deduce the generalizability of the findings to other groups and races. There is a need to expand cross-racial research to reduce health inequalities. And the reproducibility of the study makes the results more credible and more appropriate for generalization. We included only a small number of risk factors for CVD in our analysis, without in-depth studies on mediating mechanisms, which inevitably missed some possible potential associations. Finally, the exploration of adverse effects was limited to a small number of CVDs and was not validated for systemic diseases and symptoms. Because the effects of drugs on their targets are complex, many off-target effects cannot be explored by MR analysis, so further exploration through subsequent basic and clinical trials conducted in a comprehensive cross-ethnic manner is needed.

## Conclusion

The large-scale MR analysis in the present study revealed the effects of six druggable genes on hemorrhagic stroke incidence. TNFSF12 promoted IA and SAH, SLC22A4 increased the risk of IA rupture leading to SAH, and SPARC was a risk factor for ICH, for which hypertension may be a potential mediator. In addition, certain ischemic stroke side effects that may result from the inhibition of these targets are also suggested. In conclusion, our study provides genetic evidence for drug targets, and further experiments are needed to assess the effectiveness and safety of these agents.

## Supplementary Information

Below is the link to the electronic supplementary material.

Supplementary file1 (XLSX 20665 KB)

## Data Availability

The genetic data used in this study are all previously publicly accessible GWAS datasets. The data analyzed and obtained in this study process are available in the supplementary data or in the database repositories provided in the original literature.
